# Supplementary figures and images for: A comprehensive analysis identified an autophagy-related risk model for predicting recurrence and immunotherapy response in stage I lung adenocarcinoma
Source: PeerJ. 2025 May 2;13:e19366. doi: 10.7717/peerj.19366 (PMC12051938; doi:10.7717/peerj.19366)

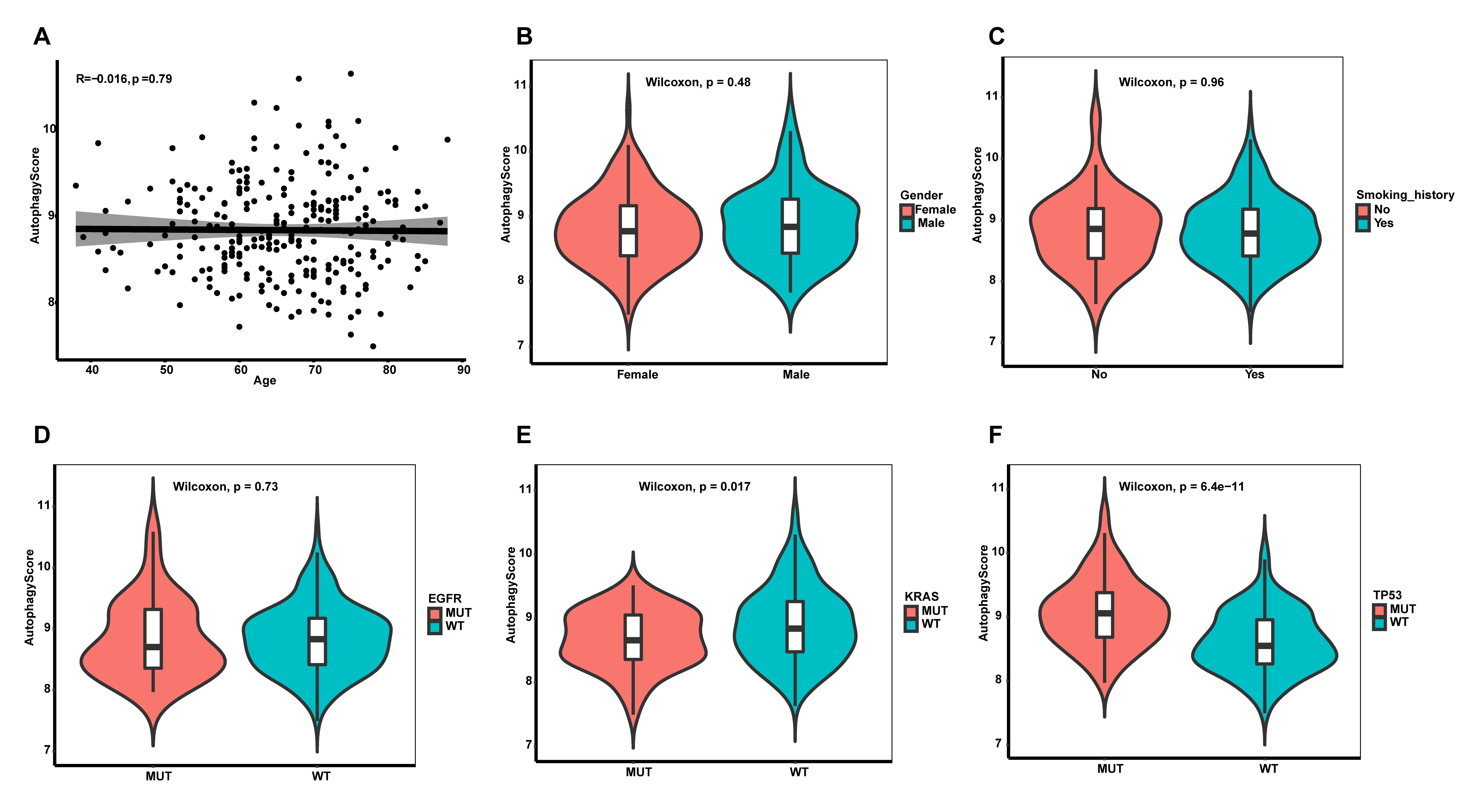

Supplement: Supplemental Information 4 [file peerj-13-19366-s004.tif]
