# Supplementary material for: A comprehensive analysis identified an autophagy-related risk model for predicting recurrence and immunotherapy response in stage I lung adenocarcinoma
Source: PeerJ. 2025 May 2;13:e19366. doi: 10.7717/peerj.19366 (PMC12051938; doi:10.7717/peerj.19366)
Supplement: Supplemental Information 5 [file peerj-13-19366-s005.docx]

**Translation for Data_acquisition**

定义自噬相关基因

Define autophagy-related genes

取交叉

Get intersecting genes

韦恩图

Venn diagram

定义颜色

Define colors for each part

从内置色板Set3中选出4个颜色使用

Select 4 colors from the built-in color palette Set3 for use.

韦恩图的名字

The name of the Venn diagram

边框线宽度

The width of the border line

标签字体

Label font

圈的颜色

The color of each circle

对应每个圈的颜色，有几个数据集，就需要有相应数量的颜色

Corresponding to the color of each circle, for the number of data sets there are, the same number of colors is required.

透明度

Transparency

此处设置每个数据集的名称颜色，也可以使用c（）函数输入颜色

Set the name color of each data set here. You can also use the c() function to input the colors.

每个区域label名称的大小

The size of the label name for each region

标签字体大小

The size of the label font

获取I期LUAD数据

Obtain the data of stage I LUAD

选取生存期完整的I期患者临床数据

Select the clinical data of stage I patients with complete survival periods.

选取相应的表达矩阵

Select the expression matrix.

**Translation for Model validation**

exp matrix

表达矩阵

ROC curve evaluate model

ROC曲线评价模型

Calculate the AUC value and its confidence interval

计算AUC值及其置信区间

Draw the time-dependent ROC curve

绘制time-dependent ROC曲线

modify the plot

美化图
